# A Data-Centric Approach for Health Care and Research in a Health Knowledge Management Platform: Implementation and Requirement-Based Evaluation Study

**DOI:** 10.2196/83608

**Published:** 2026-04-30

**Authors:** Björn Schreiweis, Benjamin Kinast, Hannes Ulrich, Tobias Bronsch, Ann-Kristin Kock-Schoppenhauer, Björn Bergh

**Affiliations:** 1 Institute for Medical Informatics and Artificial Intelligence, Kiel University and University Hospital Schleswig-Holstein, Arnold-Heller-Straße 3, Kiel, 24105, Germany, 49 0431500 ext 31601; 2 Medical Data Integration Center, University Hospital Schleswig-Holstein, Kiel/Lübeck, Germany; 3 Institute for Medical Biometric and Statistics, Section for Clinical Research IT, Universität zu Lübeck and University Hospital Schleswig-Holstein, Lübeck, Germany

**Keywords:** clinical data repository, data integration, knowledge management, interoperability, secondary use, requirements engineering

## Abstract

**Background:**

In the evolving landscape of health care, data use plays an ever-increasing role in health care IT. However, data are often siloed and uncoded free text distributed across several IT systems. This paper introduces a health knowledge management platform, designed to integrate, harmonize, and enable reuse of health care and medical research data. The platform aims to bridge the gap between research and patient care, showcased through real-world scenarios, emphasizing data harmonization and knowledge management within a health care institution. The study is based at the University Hospital Schleswig-Holstein.

**Objective:**

The main objective of this project is to design, implement, and evaluate a knowledge management platform that integrates health care and biomedical research to support use cases in both domains.

**Methods:**

The study describes the “health knowledge management platform” designed to access and gain knowledge from health care and medical research data. We performed several rounds of focus groups with stakeholders to elicit the platform requirements. In the process, we identified key aspects of the platform. From the functional requirements, we designed an architectural concept. The platform evaluation follows the Framework for Evaluation in Design Science Research and International Organization for Standardization/International Electrotechnical Commission (ISO/IEC) 25010 standard with a focus on key aspects identified and real-world scenarios. Two application scenarios, cardiology and radiology, are selected for a requirement-based, qualitative evaluation.

**Results:**

We show that our health knowledge management platform is capable of integrating diverse data formats like Health Level 7 Version 2 messages, CSV exports, and Digital Imaging and Communications in Medicine. It currently integrates over 46 million admit, discharge, transfer messages, 38 million imaging studies, and structured clinical data for approximately 1.5 million patients. The platform supports different scenarios based on its 5-layer architecture, including a clinical data repository and services like Master Patient Index and Consent Management. The evaluation against 39 predefined functional requirements showed our platform’s capability in certain real-world scenarios of cardiology and radiology. Our evaluation demonstrates that the platform covers the majority of the identified requirements to support knowledge management in health care institutions.

**Conclusions:**

Our requirement-based evaluation of the health knowledge management platform at University Hospital Schleswig-Holstein reveals its capabilities, which is possibly leading to better knowledge transfer between patient care and research. The platform’s architecture and standardized data improve the quality of data and facilitate access to knowledge. Ongoing development and potential quantitative measures will further enhance its applicability in dynamic health care landscapes.

## Introduction

In modern health care, data play a crucial role in supporting clinical decisions, improving treatment strategies, and enabling clinical research. Clinicians and researchers require access to reliable, structured, and machine-readable data for applications such as clinical decision support systems (CDSS), artificial intelligence (AI), and federated learning models [1-4]. However, in many hospitals, data remain siloed and poorly coded and exist often in free-text formats within the hospital information systems. These silos limit data reuse, complicate integration, and limit the deployment of intelligent systems that depend on structured and consistent inputs [5-7]. Additionally, the coexistence of multiple interoperability standards adds to the complexity of data integration and management [8,9].

However, instead of focusing only on technical interoperability between systems, hospitals need platforms that structure, harmonize, standardize, and make data usable across various scenarios [10]. Structured and centrally available data are a central prerequisite for building learning health care systems [11], which aim to improve patient care and research at the same time. Such systems rely on data from diverse sources, such as clinical information systems (CIS), picture archiving and communication systems (PACS), and laboratory information systems (LIS), or data from medical devices, wearable sensors, and mobile apps [12,13]. It is not sufficient to exchange data between systems. Data must be transformed into usable knowledge. This highlights the importance of knowledge management in health care. It shifts the focus from isolated data handling toward creating value from data by organizing, linking, and making it accessible for different stakeholders via an integrated platform. “Knowledge Management is therefore a conscious strategy of getting the right knowledge to the right people at the right time and helping people share and put information into action in ways that strive to improve organizational performance” [14]. This definition shows that the value of data lies not only in its availability but also in its structured, contextualized, and accessible form. There are different kinds of knowledge, but for now, we mainly focus on empirical knowledge. To implement knowledge management in health care settings, a technical infrastructure is needed that ensures interoperability, data harmonization, and support for various applications on a consistent data basis.

The Medical Informatics Initiative funded by the German Federal Ministry of Research, Technology and Space aims toward reliable and fast access to data-centric platforms for biomedical research by setting up (medical) data integration centers (Medical Data Integration Center or Data Integration Center) at the German university hospitals [15,16]. Our approach goes beyond pure secondary use of data by aiming to support both research and immediate patient care. To achieve this, we iteratively develop a health knowledge management platform architecture that not only facilitates structured data integration but also provides interfaces and functionalities, which allow the reuse of data for, for example, clinical decision support and cohort exploration. In doing so, it contributes to a future-ready infrastructure for emerging applications such as AI-driven analytics, personalized medicine, or federated learning.

The main objective of this project is to design, implement, and evaluate a knowledge management platform that integrates health care and biomedical research to support use cases in both domains. Therefore, the three subobjectives are (1) to identify the requirements of such a knowledge management platform, (2) to design an architectural concept for the knowledge management platform based on the requirements, and (3) to implement and qualitatively evaluate the platform against the requirements.

## Methods

### Ethical Considerations

The referenced studies were carried out in adherence with the Declaration of Helsinki and approved by the local medical ethics committees of Kiel University (approval B279/18 and D596/22). Informed consent was obtained from all of the participants in the studies.

### Overview

Our methodology is based on the Framework for Evaluation in Design Science Research (FEDS) by Venable et al [17], which describes the process from defining the problem, through deriving a solution based on requirement engineering (RE), evaluating the platform, and communicating the outcomes.

### Framework for Evaluation in Design Science Research

The FEDS consists of 4 components: problem, solution, evaluation, and communication.

#### Problem

The first step involves identifying and understanding the challenges related to siloed and insufficiently structured and coded health care data in a university hospital setting. Much of the clinical information is documented in unstructured formats such as free text or PDF documents. These are often embedded in hospital information systems, which limits accessibility and reuse [17]. Additionally, relevant data are distributed across various primary source systems, complicating semantic interoperability and secondary (research) use. Moreover, structured and centrally accessible data are essential prerequisites to enable future applications such as AI-based decision support or federated learning approaches [18].

#### Solution (via RE)

##### Overview

The second step of the FEDS focuses on designing and developing an effective artifact to address the identified problems. In the FEDS context, an artifact can refer to a tangible or intangible entity—such as software applications, system architectures, databases, protocols, guidelines, or frameworks—created or modified as part of the solution development process [17]. In this study, the solution is our health knowledge management platform, designed to integrate structured and unstructured, alphanumeric, and multimedia health care data while harmonizing them. The platform is further designed to improve interoperability, thereby creating a unified data management environment for both health care and research-related applications. Therefore, the development was preceded by a systematic RE process to capture and formalize the platforms’ functional needs.

##### RE Process

We used a structured RE approach to define the platform architecture [19,20]. From 2019 to 2024, we conducted multiple focus groups with a total of 27 stakeholders, covering a broad spectrum of roles and expertise:

- Strategic and technical leadership: including the chief digital officer and the heads of the Medical Data Integration Center.
- Technical and domain experts: such as specialists in metadata management, data integration, interoperability standards (eg, Health Level 7 [HL7] Fast Healthcare Interoperability Resources [FHIR], openEHR, and Digital Imaging and Communications in Medicine [DICOM]), and terminologies (eg, Systematized Nomenclature of Medicine—Clinical Terms, *International Classification of Diseases and Related Health Problems, 10th Revision, German Modification* [*ICD-10-GM*], Anatomical-Therapeutic-Chemical, and Logical Observation Identifiers Names and Codes [LOINC]).
- User representatives: including pharmacologists, an anesthesiologist, biobank directors, and data privacy specialists.
- Scenario-specific stakeholders: such as cardiologists, radiologists, a neurologist, a nephrologist, and a sleep medicine specialist who acted as product owners for the real-world evaluation.

The interdisciplinary composition of the focus groups ensured alignment with strategic goals and operational needs. Iterative discussions and repeated validation reasonably support the assumption that thematic saturation is achieved, in line with the approach described by Vasileiou et al [21].

The discussions were guided by the central question: “Which functions should the knowledge management platform provide to support health care and biomedical research?” [22,23]. Session results were summarized in a mind map (Multimedia Appendix 1) and clustered into thematic areas: data management and persistence, architecture, Findable, Accessible, Interoperable, Reusable principles [24], AI, data and information extraction, health telematics and regional integration, integration of research and patient care data, general knowledge integration, and integrated frontends.

Using this structure, we mapped each of the identified key aspects to functional requirements, which we derived from a previously created requirements catalog [25]. Additional requirements were specified using International Organization for Standardization (ISO) 29148 syntax ([Subject] [Action] [Constraint of Action]) [19], for example, “The platform must integrate data.” Possible functionalities or solutions are annotated in the respective requirements notes (Multimedia Appendix 1). Disagreements during the requirements elicitation process were resolved through moderated discussions with the RE expert.

Subsequently, we mapped these requirements to the HiGHmed architecture [15] and extended the existing layer model to define our five-layer architecture: (1) source layer, (2) staging layer, (3) normalization layer, (4) data access layer, and (5) application layer, in line with the principle of separation of concerns [26].

### Evaluation

The evaluation component involves a requirement-based artifact evaluation of the platform against the identified requirements. The primary focus lies on evaluating the functional aspects identified in the RE process. While detailed benchmarking of nonfunctional qualities (eg, performance or scalability) is beyond the current scope, quantitative insights (eg, volume of integrated laboratory data) are included. We use a technical artifact evaluation strategy guided by the quality model defined in International Organization for Standardization/International Electrotechnical Commission (ISO/IEC) 25010 [27], part of the ISO/IEC 25000 SQuaRE framework. This allows us to focus on relevant quality attributes such as interoperability, reusability, data consistency, and usability (see Key Aspects of the Platform section). The evaluation is accompanied by 2 real-world scenario-based assessments, each chosen for its distinct technical requirements:

- Cardiology: This scenario evaluates the platform’s ability to integrate and harmonize structured and unstructured clinical routine data from hospital IT systems (eg, CIS, PACS, LIS, Electronic Prescribing and Medicine Administration [ePMA], and patient-generated health data [PGHD] from mobile apps and wearables for patients with heart failure patients, with a focus on technical integration, interoperability, and traceable data reuse).
- Radiology: This scenario evaluates the platform’s functionality in processing imaging metadata and integrating demographic data to enable consent-compliant cohort identification and follow-up planning, focusing on system-level integration and traceability.

To operationalize the qualitative scenario evaluation, we applied different evaluation activities depending on the nature of the requirement and its implementation status. These activities were selected to provide traceable evidence for requirement fulfillment, in line with the ISO/IEC 25000 standard framework. Specifically, the following evaluation activities were used:

- Scenario walkthroughs: used to evaluate end-to-end functionality under real-world conditions.
- System and architecture inspections: used to verify data flows, persistence architecture, and interoperability aspects.
- Log inspections and data audits: applied to confirm the integration of structured and unstructured data (eg, Health Level 7 Version 2 [HL7 V2] and DICOM).
- Conceptual design reviews: used for requirements that have been specified, modeled, or architecturally prepared but are not yet in operational use.

These activities support a systematic evaluation of how well the platform meets the functional requirements derived from the RE process. All evaluation activities were conducted by the institution’s Data Integration Unit, a dedicated team of medical informatics experts, data engineers, and platform architects. An additional evaluation scenario is provided in Multimedia Appendix 2.

### Communication

The final step involves disseminating results to relevant stakeholders. In our case, we communicate findings through this study and use them internally to inform researchers and clinicians to promote the platform and its functionalities.

Following these structured phases ensures that the platform’s development and evaluation align with its goal of enabling interoperability and the effective health care data use across multiple domains by leveraging data integration as a central strategy.

## Results

### Problem Statement

With more than 500,000 encounters (approximately 100,000 inpatients and approximately 400,000 outpatients) annually and about 17,000 employees, University Hospital Schleswig-Holstein (UKSH) is one of the largest university hospitals in Germany and the only hospital providing maximum medical care in the state of Schleswig-Holstein (Northern Germany) [28]. It operates across 2 locations, Kiel and Lübeck, in partnership with the Medical Faculty of Kiel University and the Medical Section of the Universität zu Lübeck, forming a central hub for academic medicine in Northern Germany.

The current IT system landscape at UKSH consists of different types of information systems. The CIS builds the core of the IT system landscape, provides a general overview of the patient’s history, and serves as a basis for documentation and communication across health care domains. In addition, there are specialized department-specific systems, such as radiology information systems (RIS) or echocardiography systems in cardiology. Finally, there are the function-specific specialized systems like LIS and ePMA that support specific workflows or tasks independent of the respective department. The existence of these various systems challenges data integration, harmonization, and coding and requires careful attention to ensure that all necessary information is captured and managed appropriately. The IT system landscape and its limited semantic interoperability between the different systems often result in patient data spreading across multiple systems, sometimes even redundantly. This fragmentation complicates timely access to required information and poses challenges for researchers who require timely access to relevant data, ultimately hindering effective data use and analysis.

Moreover, there is a client separation in health care, with patient care and research operating independently from an IT and data perspective. Typically, clinicians are responsible for treating patients and have access to patient-level data for that purpose only. The processes and IT systems used in patient care are often disjoint from those used for clinical and biomedical research. Accordingly, it is inherent in the nature of these IT systems that they are primarily designed for patient care purposes rather than research, that is, supporting patient-level data access, but not cohort-level access. The organizational separation between patient care and research is partly also mandated by regulation, requiring researchers to obtain patient consent and adhere to additional processes to access the necessary data. As a result, clinicians researching on patient data either request a data export from the clinical IT service provider or manually extract the data via the applications’ user interfaces (ie, redundant data entry). Both are time-consuming, highly inefficient, and prone to transmission errors, leading to potential inaccuracies in the data and generating irreproducible research findings. Data exports are often raw CSV files per IT system, which requires manual data linkage and transformations to ensure consistency in units of measurement, as well as annotations with appropriate terminologies before allowing effective data analysis. In addition to the client separation, there is also a significant lack of automatic knowledge transfer between patient care and research. However, there is a demand in patient care for access to the latest scientific discoveries and insights to inform and improve clinical decision-making.

### Solution

#### Key Aspects of the Platform

The focus groups conducted during the requirements elicitation process identified key aspects mandatory for a knowledge management platform. The first set of key aspects relates to the scenarios the knowledge management platform needs to support, which span 3 domains: health care, research enablement, and research or secondary use. For health care, participants emphasized the need to visualize heterogeneous data types (eg, laboratory data, medication data, vital parameters, reports, images, and biosignals). Requested features included patient trajectories and the comparison of patients with similar characteristics. The integration of CDSS and AI, in general, for functionalities like smart alerting was also considered essential. A further requirement, for both scenarios, health care and research, was the inclusion of PGHD, patient-reported outcome measures, and patient-reported experience measures, with patients also having access to their own health data.

For research enablement, three key aspects were mentioned: (1) self-service feasibility analysis based on cohort definitions, (2) prescreening support for clinical trial recruitment, and (3) cohort exploration tools.

In terms of secondary use, the platform should enable care-connected research, such as research registries, by reducing redundant documentation and enabling seamless reuse of health care data. Researchers should be able to access data directly for analysis purposes or receive data exports.

After defining scenario-based needs, the view of the focus groups shifted to the data perspective. The platform needs to support various data types including structured and unstructured text, streaming data, omics data, and multimedia objects (DICOM and non-DICOM). In addition to clinical IT systems, other data sources must be integrated, including electronic health records, biomaterial information, and research data (eg, Clinical Trial Management System [CTMS]). Prefilling electronic case report forms in the CTMS with care data and integrating research data back into care are desirable. Furthermore, access to data from public sources (eg, literature databases) is requested. The platform should persist raw data on premises to serve as a single point of truth, equipped with a searchable index for data usability from the beginning.

Finally, the security and functional optimization were discussed. A clear separation of concerns is required—distinguishing between fully identifiable data, pseudonymized data, and restricted-use datasets. Raw data must be securely stored and accessible only to a small administrative group. Semantically enriched and pseudonymized datasets free from direct identifiers should be made available for health care applications and research queries. Exported data should be pseudonymized or, if possible, anonymized per project or cohort, enabling external access, for example, via national portals like the German Portal for Medical Research Data [29].

Finally, focus group participants emphasized that end users, for example, health care professionals, researchers, and lecturers, would not prefer to access data directly, but through scenario-specific applications that provide tailored visualizations and functionalities.

#### Requirements

A total of 39 requirements were organized into 5 distinct layers for the envisioned platform architecture to ensure a structured and modular approach. Specifically, 9 (23%) requirements were assigned to the source layer, 4 (10%) to the staging layer, 15 (39%) to the normalization layer, 2 (5%) to the data access layer, and 9 (23%) newly identified requirements to the application layer. A detailed overview of all 39 requirements and their assignment to the architectural layers is provided in Multimedia Appendix 2.

#### Description of the Knowledge Management Platform

**Figure 1.**
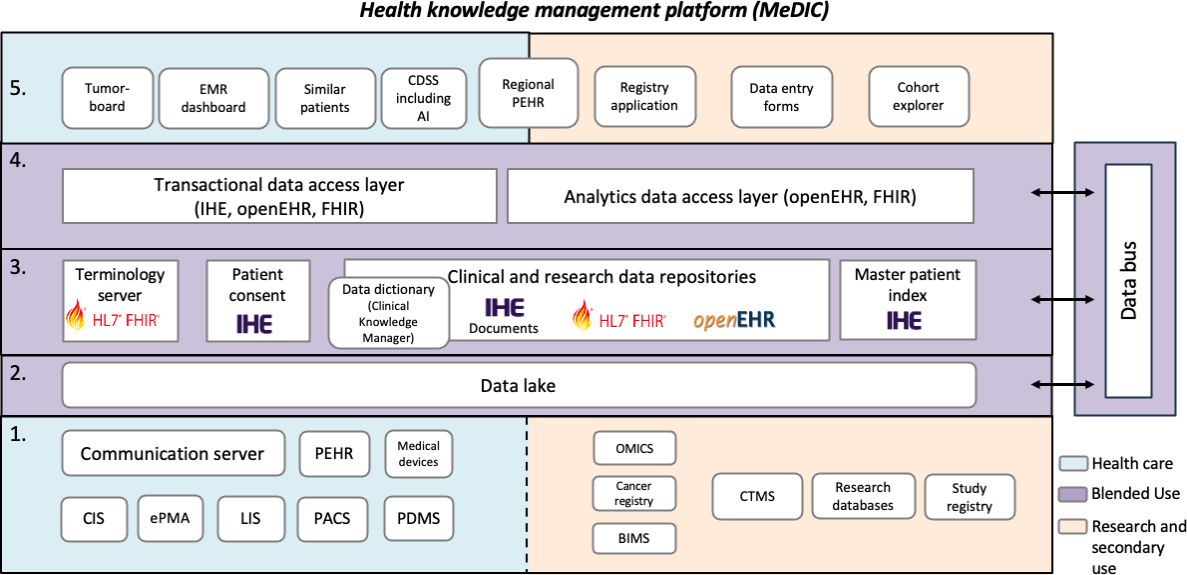
Architecture of the health knowledge management platform of UKSH MeDIC with its five layers: (1) source layer, (2) staging layer, (3) normalization layer, (4) data access layer, and (5) application layer. BIMS: Biomaterial Information Management System; CIS: clinical information system; CTMS: Clinical Trial Management System; EMR: electronic medical record; ePMA: Electronic Prescribing and Medicine Administration; FHIR: Fast Healthcare Interoperability Resources; HL7: Health Level 7; IHE: Integrating the Healthcare Enterprise; LIS: laboratory information system; MeDIC: Medical Data Integration Center; PACS: picture archiving and communication system; PDMS: patient data management system; PEHR: personal cross-enterprise health record; UKSH: University Hospital Schleswig-Holstein.

The knowledge management platform consists of 5 layers and a data bus: source, staging, normalization, data access, and application layer (Figure 1).

##### Source Layer

The source layer contains all health care and research IT systems from which data are integrated (DA-01; see Key Aspects of the Platform section). Health care systems include CIS, LIS, ePMA, PACS (including multimedia data from radiology, cardiology, neurology, and gynecology; DA-03), patient data management system, personal cross-enterprise health record (PEHR; DA-07 and DA-08-3), and medical devices. Research systems include a clinical cancer registry and tumor documentation system, a Biomaterial Information Management System, a CTMS, systems for generating and analyzing OMICS data, research databases, and a local study registry. The CTMS supports the data entry process in clinical trials, but also the management of study appointments, participants, and electronic case report forms. The local study registry contains all studies running at USKH including eligibility criteria to support the prescreening for clinical trials in the future. These studies can be integrated from national and international study registries like ClinicalTrials.gov or drks.de (DA-27) [30]. Data are transferred via interfaces (eg, HL7 V2) and a communication server to the data lake (see Staging Layer section; DA-02) or exported and stored consistently into the data lake (DA-01-4). Data types range from structured (eg, laboratory reports; DA-01-4) to semistructured and free-text (eg, discharge letters; DA-01-3) and multimedia data (eg, computed tomography [CT] images; DA-03).

##### Staging Layer

The staging layer consists of a central data lake with analytical processing capabilities (DP-02) [31], supporting both batch and message-based (streaming) data (DP-07). Data flows are orchestrated using Apache NiFi. Incoming data are first persisted in S3 buckets (DS-03) to ensure reliability and data provenance (DS-02-2). A Master Patient Index Identifier is requested from the Master Patient Index (MPI) in the normalization layer and replaces the hospital’s patient identifier in all processed records or data (DP-20-3, DP-20-5, and DP-21). Subsequently, data are indexed in Elasticsearch to enable searchability and discovery (DS-13) and forwarded via the data bus. Unlike the source layer, the staging layer processes both health care and research domains (blended use).

##### Data Bus

The data bus is built on Apache Kafka and enables scalable, topic-based data distribution for both health care and research data [32]. Topics may contain either full data records or references (eg, images or electrocardiography [ECG] paths) for downstream retrieval.

##### Normalization Layer

The normalization layer provides harmonization and standardization of data from internal (eg, CIS) and external sources (eg, PEHR; DP-19). It includes the MPI, Patient Consent Manager (PCM), clinical and research data repositories, and a terminology server, based on interoperability standards, including HL7 FHIR, openEHR, DICOM (DP-23), and Integrating the Healthcare Enterprise (IHE). Patient identity management is handled via the MPI (DP-20-3 and DP-20-5) compliant with IHE Patient Identifier Cross-Referencing [33] and IHE Patient Demographics Query (DSC-04) [34]. The PCM manages and distributes versioned broad consents to authorized downstream systems (DAS-13) [35]. Patient-referenced metadata (DP-21) are stored in an openEHR-based clinical data repository (DS-02-2, DS-05, DS-08, and DP-33-1), while nonpatient-referenced data, such as study metadata or data about drugs, are maintained in a FHIR-based repository (DS-02-2, DS-05, DP-06, and DP-33-1). OpenEHR compositions may additionally be persisted as IHE Cross-Enterprise Document Sharing (XDS) documents (DP-33-1). The CDR is queried via openEHR Archetype Query Language (AQL), and its data models are openly accessible and managed in a clinical knowledge management tool (MDM-01) [36], while FHIR models are provided via Simplifier [37].

The terminology server supports mapping of local codes to standardized terminologies such as LOINC (eg, for laboratory data), *ICD-10-GM*, and Systematized Nomenclature of Medicine—Clinical Terms (DP-01). It provides versioned terminologies and FHIR ConceptMaps to ensure semantic interoperability across systems (DP-01) [38].

##### Data Access Layer

The data access layer includes transactional and analytical interfaces. The transactional data access layer supports persistence and retrieval via openEHR, FHIR, and IHE-based application programming interfaces, while the analytical data access layer supports cohort-based queries and research-driven analysis (DAS-03). Data exchange between the data lake, CDR, and project-specific data marts is handled via the data bus (DAS-12).

##### Application Layer

The application layer provides end-user applications that access clinical and research data via the data access layers. In the health care domain, this includes a Molecular Tumor Board (MTB) application that supports preparation for MTB sessions by providing access to patient-level data including imaging, clinical, and OMICS data (DR-01 and DR-03-3) [39]. An Electronic Medical Record Dashboard offering unified patient-level views (DR-01, DR-03, and DR-04) and a “Patients like mine” similarity application (DR-03-1, DR-03-2, and DR-03-3) [40,41] are planned, as well as various CDSS including AI-based tools. A regional PEHR application (DA-07) to integrate PGHD from smartphones, wearables, body sensors, and self-reported data via FHIR (DA-18) was tested and evaluated in a proof-of-concept. Research-oriented applications include structured data entry forms (DR-01), registry integration (DR-01) [42], and cohort explorer for feasibility analysis (DR-02) and data requests (DR-01 and DR-05). Access to data based on use requests is governed via consent management (DAS-13 and DSC-04). A centralized user management system is integrated with the hospital identity management system (DSC-12).

### Evaluation

#### Overview

The evaluation assesses the platform against the previously identified functional requirements. We conducted scenario-based evaluations in 2 distinct medical domains to cover a broad range of technical and research-related requirements. The primary results from the cardiology and radiology scenarios are presented below, with an additional neurology scenario provided in Multimedia Appendix 2.

#### Clinical Routine Data in a Cardiology Scenario

The cardiology scenario evaluates the platform’s technical integration, traceability, and reuse of structured clinical data for patients with heart failure, integrating routine care data [15]. The evaluation focuses on system-level interoperability and requirements fulfillment rather than on clinical effectiveness, patient outcomes, or therapeutic impact.

Patient data were processed under a study-specific consent, covering electronic medical record reuse (ethics approval obtained at Kiel University B279/18). Clinical data from CIS, LIS, and ePMA were integrated (DA-01). Demographic, laboratory, and anamnesis data (DA-01-2 and DA-01-3) were ingested as HL7 V2 admit, discharge, transfer (ADT) and observation result unsolicited (ORU) messages from the hospital’s communication server (DA-01 and DA-02), while medication data were imported via CSV exports. Data were persisted in the staging layer (DS-13) and semantically harmonized using standardized terminologies such as LOINC, Anatomical-Therapeutic-Chemical, and *ICD-10-GM* (DP-01). Follow-up and echocardiography data were captured via openEHR-based data entry forms (DR-01) [43,44]. Cross-system record linkage and separation of identifying and medical data were ensured via the MPI (DP-20-3, DP-20-5, and DSC-04), with metadata stored for traceability in the openEHR-based CDR (DS-02-2 and DS-03), including the technically implemented ingestion of PEHR-derived structured data (DA-08-3) as a proof-of-concept. In addition, sensor-derived PGHD, including heart rate and activity measurements recorded via wearable devices, were transmitted as FHIR resources from a mobile app to the PEHR and ingested into the platform (DA-18, DA-07, and DA-08‐3) and linked via the MPI (DP-21 and DP-19) as a technical proof-of-concept.

Finally, structured data corresponding to metadata from 710 patients were transformed into validated openEHR templates (DP-06, DP-23, and DP-33-1) and persisted in the openEHR-based CDR (DA-01-4, DS-05, DS-08, and DP-21) [43-47], derived from more than 38,000 HL7 messages (DA-01, DA-02, and DP-07). All openEHR templates were validated with clinicians and published (DP-23) [36]. The resulting dataset was queried using AQL and exported as a CSV file for analysis (DAS-12 and DR-05).

Thus, the scenario meets 26 of 39 (67%) platform requirements. The evaluation activities and corresponding evidence at the requirements level are detailed in Table 1. A consolidated assessment of the resulting quality attributes according to ISO/IEC 25010 across both scenarios is summarized in Table 2.

**Table 1.** Alignment of platform requirements with scenarios.

Requirement ID	Layer	Description	Implemented in platform	Evaluation activity	Evidence or outcome
DA-01	SrcL^a^	The platform must acquire data from different heterogeneous source systems.	✓^b^	Scenario walkthrough (cardiology, radiology)	Successful ingestion of HL7 V2^c^ (ADT^d^/ORU^e^) DICOM^f^, and CSV data from CIS^g^, LIS^h^, and PACS^i^ persisted as records in the data lake
DA-01‐2	SrcL	The platform must acquire structured alphanumeric EHR^j^ data.	✓	Log inspection (cardiology)	2902 laboratory reports acquired from HL7 V2
DA-01‐3	SrcL	The platform must acquire unstructured alphanumeric EHR data.	✓	Cardiology scenario	1016 anamnesis forms acquired
DA-01‐4	SrcL	The platform must acquire data from heterogeneous sources in a consistent manner.	✓	Technical architecture inspection	HL7 V2, CSV, FHIR^k^, and DICOM processed through standardized ETL^l^ pipelines and stored in uniform data structures within the data lake and CDR^m^ (verified via ETL configuration and storage inspection)
DA-02	SrcL	The platform must acquire structured data based on interoperability standards from the IT and health care domains.	✓	Scenario walkthrough	Incoming data received via HL7 V2 (ADT, ORU, BAR^n^) messages from, eg, CIS and LIS
DA-03	SrcL	The platform must acquire multimedia data from various domains.	✓	Radiology scenario; ingestion test	CT^o^ DICOM successfully retrieved from PACS (including data from, eg, radiology, cardiology, neurology, and gynecology) and stored in the platform [48]
DA-07	SrcL	The platform must acquire data from cross-institutional sources.	✓	Cardiology PGHD^p^ ingestion in PEHR^q^ (technically implemented)	FHIR-based PGHD integration pipeline from external medPower to PEHR to the platform, verified by successful ETL execution and persisted openEHR compositions in proof-of-concept
DA-08‐3	SrcL	The platform must acquire data from personal health records with all of the following nature: * structured * unstructured.	✓	Cardiology PGHD workflow	Structured FHIR resources generated from Apple HealthKit; transmitted via PEHR, transformed into openEHR compositions, and stored in CDR (verified by stored compositions in proof-of-concept)
DA-18	AppL^r^	The platform must acquire data from (FHIR-based) apps.	✓	Cardiology scenario	Successful ingestion of FHIR data from HealthKit (Apple Watch) via medPower and PEHR; ETL implemented to map to openEHR in proof-of-concept
DA-27	SrcL	The platform must integrate data from public sources.	TBD^s^	Conceptual design review	Functionality not yet implemented due to regulatory prerequisites
DP-07	SL^t^	The platform must process data from data streams.	✓	Cardiology scenario	HL7 V2 data streams (ORU, ADT, and BAR) processed weekly
DS-02‐2	SL	The platform must store both data and metadata from multiple sources in a single storage.	✓	System inspection	HL7^u^, DICOM, and CSV data including metadata, persisted in S3 storage and indexed via Elasticsearch (verified by storage inspection)
DS-03	SL	The platform must store the original data.	✓	System inspection	Original HL7 and CSV files stored in S3 buckets for traceability (verified by storage inspection)
DS-05	NL^v^	The platform must store data in a data repository.	✓	System inspection	Structured data stored in openEHR-based CDR; ~1.5 million patient records integrated (verified by storage inspection)
DS-08	NL	The platform must store the data in a way that it is available in a clinical data repository.	✓	Scenario execution (all)	AQL^w^ queries executed on CDR returned structured longitudinal patient data, enabling cohort identification in both scenarios (verified via query results)
DS-13	SL	The platform must store data in all of the following ways: * full-text indexed * searchable.	✓	Technical inspection	Full-text indexing via Elasticsearch; supports full-text search (verified via query results)
DP-01	NL	The platform must process data by mapping it to multiple terminology standards.	✓	Cardiology scenario	LOINC^x^, ATC^y^, and ICD-10-GM^z^ applied during ETL for laboratory, medication, and diagnosis data (verified via query results)
DP-02	NL	The platform must process unstructured data by using natural language processing (NLP) methods.	TBD	Conceptual design review	NLP functionality conceptually designed but not implemented
DP-06	NL	The platform must process all of the following: * data * metadata.	✓	System design inspection	Metadata extraction from HL7, DICOM, CSV; structured storage of both in openEHR CDR (verified via query results)
MDM-01	NL	The platform must provide metadata management (tools).	✓	Scenario execution (radiology)	Imaging metadata stored, searchable via DICOM tag filters (verified via query results)
DP-19	NL	The platform must link external data with local data.	(✓)^aa^	Conceptual design review	PGHD from patients linked via MPI to clinical data in proof-of-concept
DP-20‐3	NL	The platform must process data by linking it to at least 1 of the following Master Patient Indexes: * institutional * cross-institutional.	✓	Scenario execution (all)	MPI links data across systems; contains 2.3 million patient identities
DP-20‐5	NL	The platform must process data by using at least 1 of the following IDs: * locally unique record ID * locally unique case IDs.	✓	Scenario execution (all)	Local patient IDs used in MPI, mapped to MPI-ID for further identification
DP-21	NL	The platform must process data to link records from various source systems.	✓	Scenario execution (all)	For example, record linkage across CIS, PACS, LIS, ePMA^ab^
DP-23	NL	The platform must process data according to a specified semantic data model.	✓	Technical inspection	Structured data instances validated against openEHR archetypes and templates; conformance verified through successful persistence in the CDR without schema violations
DP-33‐1	NL	The platform must process data by transforming it into an open standardized format.	✓	Scenario execution (all)	HL7, CSV, and FHIR inputs transformed into openEHR compositions and stored in CDR; successful transformation confirmed by absence of processing errors and validated template bindings
DSC-04	NL	The platform must provide a pseudonymization ID (PID) to disconnect medical data from identifying data.	✓	Scenario execution (all)	PID service integrated; separates identifying (MPI) from clinical data (CDR)
DAS-03	AL^ac^	The platform must provide various data analysis techniques.	TBD	Conceptual design review	Framework supports integration of external analysis tools; not yet implemented
DAS-12	AL	The platform must extract datamarts for research-related analysis.	✓	Scenario execution (all)	Structured data exports to CSV; used for analytics in cardiology and radiology scenarios
DAS-13	NL	The platform must only analyze (patient-) data for which consent has been obtained.	✓	Scenario execution (radiology)	Consent status checked in PCM^ad^ before recontact; enforced before analysis
DR-01	AppL	The platform must provide user group–specific interfaces.	✓	Scenario execution (cardiology)	Data entry tool tailored for study nurses
DR-02	AppL	The platform must provide a user interface for cohort exploration.	✓	Scenario execution (cardiology)	Cohort builder enables data transfer unit to search by, eg, diagnosis, laboratory, procedure, and consent data
DR-03	AppL	The platform must provide enhanced visualization for holistic multimodal care information.	TBD	Conceptual design review	Not implemented
DR-03‐1	AppL	The platform must provide user group–specific interfaces for predictive data analysis.	TBD	Conceptual design review	Not implemented; planned for future artificial intelligence module integration
DR-03‐2	AppL	The platform must provide a user interface for the comparison of patients with similar medical histories.	TBD	Conceptual design review	Not implemented; AQL queries for patient grouping prepared
DR-03‐3	AppL	The platform must provide trajectory data representation.	✓	Conceptual design review	Longitudinal patient trajectories rendered in the Molecular Tumor Board application, displaying multimodal data on timeline (verified via system demonstration)
DR-04	AppL	The platform must provide an interface for context-related external calls from the clinical information system (CIS/EMR^ae^).	TBD	Conceptual design review	Not implemented
DR-05	AppL	The platform must allow querying qualified search results.	✓	Scenario execution (cardiology)	AQL used for structured queries across CDR; results exported for analysis
DSC-12	AppL	The platform must enable single-sign-on.	(✓)	Conceptual design review	Designed but not yet fully enabled in the live system

a SrcL: source layer.

b *✓*: fulfilled.

c HL7 V2: Health Level 7 Version 2.

d ADT: admit, discharge, transfer.

e ORU: observation result unsolicited.

f DICOM: Digital Imaging and Communications in Medicine.

g CIS: clinical information system.

h LIS: laboratory information system.

i PACS: picture archiving and communication system.

j EHR: electronic health record.

k FHIR: Fast Healthcare Interoperability Resources.

l ETL: extract-transform-load.

m CDR: clinical data repository.

n BAR: billing account record.

o CT: computed tomography.

p PGHD: patient-generated health data.

q PEHR: patient cross-enterprise health record.

r AppL: application layer.

s TBD: to be done.

t SL: staging layer.

u HL7: Health Level 7.

v NL: normalization layer.

w AQL: Archetype Query Language.

x LOINC: Logical Observation Identifiers Names and Codes.

y ATC: Anatomical-Therapeutic-Chemical.

z ICD-10-GM: International Classification of Diseases and Related Health Problems, 10th Revision, German Modification.

aa (*✓*): partially fulfilled.

ab ePMA: Electronic Prescribing and Medicine Administration.

ac AL: Access layer

ad PCM: Patient Consent Manager.

ae EMR: electronic medical record.

**Table 2.** Consolidated quality attribute evaluation.

Quality attribute	Cardiology scenario findings	Radiology scenario findings
Interoperability	Demonstrated integration of structured and unstructured data from 4 source systems (HL7 V2^a^ and FHIR^b^).	Integrated demographic and imaging metadata from PACS^c^ and CIS^d^ via CSV-based ETL^e^ processes and MPI^f^ linkage.
Data accuracy and consistency	Achieved through the use of standardized terminologies (*ICD-10-GM*^g^ and LOINC^h^) and validation of templates by clinical experts.	Ensured by automated metadata extraction from radiological systems and consistent use of patient IDs via MPI ensured reliable linkage and cohort formation.
Reusability	Extraction logic and openEHR templates were reused from more than 38,000 HL7^i^ messages (anamnesis forms: n=1016, echocardiography entries: n=616, laboratory reports: n=2902, and medication entries: n=6231).	The cohort identification process (unique patients: n=1744 and unique CT^j^ studies: n=4146) and corresponding query logic can be reused for other imaging-based follow-up studies with similar criteria.
Traceability	Enabled by storing metadata and source system information alongside clinical data, ensuring transparent data provenance.	Full provenance tracking of imaging data was achieved by retaining source system identifiers and metadata (eg, modality and scan date).
User or acceptance or usability	Manual data entry interfaces (eg, echocardiography) were cocreated with clinicians and study nurses, and the final dataset was validated with domain experts from external study sites (eg, other HiGHmed sites).	Automated generation and delivery of final patient lists to the radiology department replaced manual chart review and simplified recruitment workflows.

a HL7 V2: Health Level 7 Version 2.

b FHIR: Fast Healthcare Interoperability Resources.

c PACS: picture archiving and communication system.

d CIS: clinical information system.

e ETL: extract-transform-load.

f MPI: Master Patient Index.

g *ICD-10-GM*: *International Classification of Diseases and Related Health Problems, 10th Revision, German Modification*.

h LOINC: Logical Observation Identifiers Names and Codes.

i HL7: Health Level 7.

j CT: computed tomography.

#### Recontacting Patients in a Radiology Scenario

The radiology scenario evaluates the platform’s technical integration, traceability, and reuse of CT imaging metadata for cohort identification of patients who underwent hip or spine CT examinations (ethics approval obtained at Kiel University [D596/22]). The evaluation focuses on data integration, metadata processing, and consent-compliant record linkage (DAS-13), while diagnostic accuracy or clinical effectiveness are out of scope [49]. The platform supported cohort preparation by combining pseudonymized CT imaging data from PACS (DA-03) with demographic data from the CIS (DA-02 and DP-21) using record linkage. Eligibility criteria (age, broad consent permitting data use and recontact, CT scan type, and CT scan age) were applied via structured queries (DP-21 and DR-05). Imaging metadata (DP-06) stored in the data lake were processed within the platform (MDM-01, DS-05, and DS-02‐2) [48]. Imaging metadata and radiological reports were ingested as daily CSV reports into the S3 bucket in the staging layer (DA-01, DA-01-2, DA-01-4, DS-03, and DP-07), persisted in the data lake, and linked via the MPI (DP-20-3 and DP-20-5) [48]. Patient demographics were accessed via the MPI, and consent information was verified through the PCM prior to analysis (DAS-13, DP-20-3, and DSC-04). The CDR provided structured *ICD-10-GM* diagnoses (DS-08, DP-01, DP-23, and DP-33‐1) for eligibility filtering and querying (DR-05 and DS-13). Query results were exported as CSV datasets (MPI Identifiers, patient addresses, and vital status; DAS-12), containing 1744 unique eligible patients for follow-up processing. Subsequent patient communication was outside the platform’s scope.

The structured integration of imaging metadata and patient demographics enabled automated cohort identification and facilitated follow-up planning. Beyond the immediate gains for recruitment and research workflows, the setup also provides long-term efficiency: once implemented, additional cohorts can now be identified based on the continuously updated data within the platform. Thus, the radiology scenario fulfills 23 of 39 (59%) identified platform requirements. The evaluation activities and corresponding evidence at the requirements level are detailed in Table 1. A consolidated assessment of the resulting quality attributes according to ISO/IEC 25010 across both scenarios is summarized in Table 2.

#### Operational Assessment

In addition to the scenario-based requirement evaluation, we collected operational data metrics to provide quantitative evidence of the platform’s real-world data handling capabilities. The platform is currently connected to 10 heterogeneous clinical source systems, including ORBIS (CIS with RIS module), OPUS-L (LIS), MEONA (medication documentation), MUSE (ECG), Deep Unity (PACS), Viewpoint (gynecology and obstetrics), ODSEasy (clinical cancer registry and tumor documentation system), NEXUS (pathology information system), COPRA (patient data management system), and CentraXX (CTMS).

On a weekly basis, the platform processes approximately 180,000 HL7 ADT, 120,000 HL7 ORU, and 120,000 HL7 billing account record messages, in addition to around 30,000 DICOM studies, 12,000 RIS reports, 2500 ECG datasets, and 7000 discharge letters. The total alphanumeric data volume amounts to approximately 1 to 1.5 GB per week. As of early 2026, the platform has processed approximately 46.5 million ADT, 39.8 million ORU, 28.1 million billing account record messages, 38.3 million DICOM studies, 144,000 ECG datasets, and 4.1 million discharge letters. The MPI contains demographic records for over 2.3 million patients (since 2000), and the openEHR-based CDR stores structured data from approximately 1.5 million patients in more than 85 million compositions (including ~12 million diagnoses, ~13 million procedures, ~10 million medication requests, ~2.8 million scores from intensive care units, ~340,000 UKSH broad consents, and information on ~58,000 biospecimens). This data volume results from the platform’s integration strategy: when specific data elements are requested, they are not only integrated for the selected cohort but also systematically incorporated for all patients across legacy and ongoing data. For example, a request for anesthetics data in a specific intensive care unit in 2020 leads to the integration of anesthetic data from the introduction of the respective IT system to the present, including future records.

## Discussion

### Principal Findings

This study presents the implementation and requirement-based evaluation of a health knowledge management platform at UKSH, assessed against 39 functional requirements and 2 real-world scenarios. To overcome the traditional separation between patient care and biomedical research, the platform integrates heterogeneous clinical data (eg, CIS, PACS, and LIS) into a structured, interoperable environment [50]. Requirements were derived through stakeholder focus groups, building upon the developed requirements catalog by Kinast et al (30/39, 77%) [25]. The catalog provided a comprehensive and systematically defined foundation for key interoperability and data integration requirements, enabling efficient requirements formalization and traceable coverage. In total, 9 of 39 (23%) additional requirements were specified using ISO 29148-compliant syntax to address context-specific needs. Overall, 30 of 39 (77%) requirements are fully implemented, while the cardiology scenario fulfills 26 of 39 (67%) and the radiology scenario fulfills 23 of 39 (59%).

However, its functional scope extends well beyond the functionalities necessary to support the selected scenarios. For example, the platform integrates radiological images (eg, x-ray, CT, and magnetic resonance imaging) together with corresponding free-text reports (DA-01-3) [48], although structured extraction and storage of report content in the CDR are not yet implemented. Two requirements stay partially or not yet fulfilled: single sign-on, which is currently limited to selected applications (eg, MTB; DSC-12), and linkage of external public data sources (DP-19). The latter, including integration of regional death registries, is on hold due to legal and organizational constraints.

The integration of patient profile-based information from public sources (DP-19), for example, to support advanced decision support use cases, remains conceptually defined but not yet operational due to legal and technical constraints. Together with the incomplete single-sign-on integration (DSC-12), these gaps reflect the current implementation status rather than architectural limitations.

Our platform supports a range of data-driven applications via the data access layer, although user group-specific interfaces for predictive data analysis (DR-03-1) have yet to be developed. A key challenge is the prevalence of unstructured clinical documentation, which limits structured reuse and data quality [51]. To address this, the normalization layer is being extended with natural language processing capabilities, and an initial pipeline for allergy information extraction from discharge letters (DP-02) has been implemented [31].

An MTB application has been deployed, enabling visualization of oncological histories, patient comparison and cohort identification, and analytical functionalities (DAS-03, DR-03, and DR-03-3) [39,52]. It is also in active use to support patient care as part of the MTB meetings. Additional functionalities include end-user cohort exploration (DR-01) and data entry (DR-05) that are planned but currently restricted to specific user groups, that is, system administrators or study nurses, respectively. Our platform shares its openEHR-based foundation with the system described by Biermann et al [53], whose work on patient trajectory analysis and predictive interfaces illustrates the extensibility of this architecture. However, some applications envisioned remain on the roadmap (eg, patients like mine and end user–enabled cohort exploration). The identified gaps highlight the need for further iterative development to realize the platform’s full potential. Its design was guided by defined key aspects (see Key Aspects of the Platform section), aiming to ensure relevant quality attributes such as interoperability, reusability, data consistency, and usability aligned with the specific requirements of the UKSH environment.

A key architectural decision was the adoption of an openEHR-based CDR instead of a FHIR-based repository. While FHIR primarily supports real-time data exchange, openEHR provides a semantically rich, longitudinal data model suitable for persistent storage and consistent querying across both patient and cohort levels, posing an essential requirement for secondary use and advanced analytics [54]. Its archetype-based approach enables explicit clinical content modeling, ensuring semantic consistency and modular reuse across use cases and front ends while maintaining interoperability via FHIR interfaces. This decision is supported by the growing adoption of openEHR across European health care systems, including countries such as Slovenia [55], Sweden [56], Norway, and Finland [57] as well as major academic hospitals such as Karolinska University Hospital and University Hospital Basel [58], and the region of Catalonia [59]. The approach also aligns with the open platform principles defined by the Aperta Foundation [60], which promote openness, semantic interoperability, and modularity.

To complement this foundation, FHIR-based applications could be integrated into the application layer, enabling patients to contribute personal health data (PGHD). Conceptually, such applications could also be considered part of the PEHR within the source layer, with data integrated bottom-up via the data lake. The platform architecture and interfaces are designed to support both integration options, allowing source systems either to maintain independent persistence or to rely on the platform’s normalization layer as a shared persistence layer. We did not consider integrating the knowledge management platform directly into the CIS, as CIS platforms are primarily designed for billing, individual patient-level care, and documentation rather than cohort-level analysis or research data export. Establishing a separate platform increases architectural flexibility, without interfering with clinical workflows or billing-related functionality constraints. This also ensures that patient care remains uncompromised, as CIS and corresponding systems are not burdened with additional loads from research-related queries. Furthermore, it aligns with Findable, Accessible, Interoperable, Reusable principles by enhancing data accessibility and promoting reuse in research contexts [24].

In the platform’s initial architectural design, IHE profiles played a significant role [15]. Currently, Patient Identifier Cross-Referencing [33] and Patient Demographics Query [34] are used within the MPI, along with Advanced Patient Privacy Consents [61] for consent management. Many openEHR compositions are additionally persisted as IHE XDS-compliant documents. However, as XDS-based access retrieval is not required for current scenarios, data retrieval is performed via openEHR AQL. The architectural separation of patient identifiers, consent data, and medical content supports data protection and enables modular governance. In alignment with IHE principles, our platform manages an MPI, a consent repository, and a CDR to persist the respective data types. This modular structure enables precise permission management via fine-grained consent enforcement [35]. Currently, the platform supports consents for the research-related use of patient care data, leftover biomaterial, recontact in case of incidental findings, and recontact for future research projects (ie, recruitment and follow-up) [35]. Because the platform processes both consented and nonconsented patient data, it can also support legally authorized research models beyond informed consent, such as opt-out–based research based on a specific regulation.

### Limitations

One limitation concerns the stakeholder selection in the requirements elicitation process, which primarily emphasized functional requirements. Nonfunctional aspects such as scalability, security, performance, and usability were considered at the architectural level but not systematically elicited as explicit requirements. The platform’s modular and layered architecture follows separation of concerns principles, allowing nonfunctional requirements to be addressed within specific components. However, these aspects were not formally evaluated in this study.

Although the requirements catalog by Kinast et al [25] facilitated the formalization and data integration requirements, it did not address user interface aspects. Consequently, 9 additional requirements had to be specified separately, expanding the scope of the RE process. The RE process did not include a dedicated study-specific stakeholder analysis. However, participation in the HiGHmed consortium ensured broad clinical domain representation across multiple use cases [15]. Additional requirements were elicited through engagement with clinicians involved in the evaluated scenarios.

The platform builds on an openEHR CDR, a technology traditionally applied to electronic health records in institutional or regional infrastructures [62-64]. Our application as a knowledge management platform integrating both research and patient care contexts represents an extended use of this technology.

Structured data are a prerequisite not only for research scenarios and applications but also for decision support. However, clinical documentation still heavily relies on unstructured free-text, either entered directly into the CIS or recorded through dictation and transcription. As a result, requirements such as DR-03-1 to DR-03-3 remain a challenge, as the respective functionalities depend on structured data. This highlights the need to establish structured data as a foundation before enabling advanced care functionalities. To address this, we plan to expand the platform’s normalization layer to further include natural language processing and information extraction features, enabling the processing and structuring of the large volume of free-text reports from the CIS and its subsystems [31].

Finally, the evaluation has primarily focused on research-oriented scenarios. Broader deployment in routine care, especially for real-time decision support, remains to be assessed. Although the platform provides the necessary technical interfaces, additional application development is required to evaluate clinical integration at scale, fully demonstrating the platform’s potential in both biomedical research and patient care settings.

### Risk Assessment

Every modification of an IT system poses risks to data consistency, availability, and quality, particularly when integrating heterogeneous source systems via different interfaces [65]. Each data transformation step introduces the potential for errors, and repeated transformation increases the risk of inconsistencies between datasets. Our approach of acquiring raw data and transforming them centrally only once reduces repeated transformations but still introduces transformation risks. To mitigate these, we use automated processing, code reviews, and structured testing procedures [66]. Nevertheless, occasional errors occur. Once identified, the corresponding extract-transform-load process is corrected, and the affected data are reintegrated from the original S3 storage. Although issues in the source data itself cannot be resolved, detected anomalies can be flagged within the integrated data view.

Data reliability varies by type and depends on the original purpose of documentation. For example, laboratory measurements are typically generated through standardized processes, whereas billing-related data such as diagnoses or procedures follow regulatory and reimbursement-driven documentation logic. Platform transparency regarding data provenance and quality is therefore essential. Each newly integrated data source typically includes the complete dataset of the corresponding system, including live and legacy data, rather than cohort-specific subsets. This “integrate once, use many” strategy promotes long-term sustainability and data reuse.

Beyond technical risks, governance and long-term maintenance represent additional challenges. Sustainable operation depends on strong organizational support and compliance with data privacy regulations. At UKSH, the platform is institutionally anchored through a board resolution and supported by internal funding as well as national initiatives (Network University Medicine), which adds to its long-term stability.

### Comparison With Prior Work

Our health knowledge management platform integrates both health care and research functionalities within a unified architecture. This contrasts with many existing initiatives that focus either on specific use cases or predominantly on research-oriented data integration [67-69].

In Germany, initiatives such as the Medical Informatics Initiative and Network University Medicine [16] have significantly advanced health care data integration. As part of the HiGHmed consortium, we set the foundation for our platform by introducing open platforms and interoperability principles to enhance health care and research across institutional boundaries [15]. Building on this foundation, our platform extends these concepts by integrating both research and health care functionalities within a single integrated platform. Similarly, MIRACUM and DIFUTURE focus on cross-institutional data integration and secondary analyses to inform clinical practice and medical innovation [70,71]. While these initiatives emphasize research data use, our platform bridges the gap by unifying research and health care workflows, enabling bidirectional data reuse.

Internationally, comparable efforts exist. For instance, the Mayo Clinic Platform facilitates secure access to deidentified clinical data for research and innovation [72]. Unlike Mayo’s Enterprise Data Trust, which primarily functions as a nontransactional analytics repository, our platform additionally supports real-time clinical workflows (eg, MTB) and research data capture directly from patient care processes, enabling immediate translational use within UKSH. Similarly, the World Health Organization’s Integrated Data Platform supports large-scale population health monitoring [73] but does not integrate institutional patient care and research workflows within a unified platform.

Overall, while prior initiatives have significantly contributed to secondary use of health care data, our platform emphasizes the operational integration of health care and research through centralized consent management and a layered architecture based on separation of concerns principles.

### Conclusions

This study describes and evaluates the UKSH health knowledge management platform, demonstrating how its multistandards approach effectively addresses interoperability challenges while ensuring data consistency. The scenario-based evaluation illustrates the platform’s capability to support research based on real-world health care data. Its layered architecture and harmonization processes provide a structured data access layer that enables both clinical and research applications.

Future developments will focus on (1) expanding the application layer with cohort exploration tools and AI-supported data analysis tools [18] and (2) developing user-specific interfaces to enhance clinical usability. Hereby, we expect benefits through a reduction of manual data preparation for cohort identification, enabling more efficient study planning and data-driven health care. These applications will be developed to create a direct impact on patient care based on the partially and unfulfilled requirements, which will undergo further evaluation. Inspired by the “Ten Topics to Get Started in Medical Informatics Research” [74], our platform has the potential to improve several key areas in medical informatics, pushing the boundaries of both clinical practice and research capabilities.

## Supplementary material

10.2196/83608 Multimedia Appendix 1 Mind map of identified functional aspects. This appendix contains the structured mind map generated during the stakeholder focus group sessions. The visualization summarizes and clusters the identified functional aspects of the envisioned knowledge management platform and documents the conceptual basis for the derivation of the 39 functional requirements.

10.2196/83608 Multimedia Appendix 2 Additional scenario implemented and evaluated with the platform. This appendix contains the detailed description of the neurology evaluation scenario. It further provides the complete requirements mapping table (39 functional requirements) including architectural layer assignment and fulfillment across the cardiology, radiology, and neurology scenarios, as well as the corresponding literature references from Kinast et al.

## Abbreviations

ADT: admit, discharge, transfer
AI: artificial intelligence
AQL: Archetype Query Language
CDR: clinical data repository
CDSS: clinical decision support system
CIS: clinical information system
CT: computed tomography
CTMS: Clinical Trial Management System
DICOM: Digital Imaging and Communications in Medicine
ECG: electrocardiography
ePMA: Electronic Prescribing and Medicine Administration
FEDS: Framework for Evaluation in Design Science Research
FHIR: Fast Healthcare Interoperability Resources
HL7: Health Level 7
HL7 V2: Health Level 7 Version 2
*ICD-10-GM*: *International Classification of Diseases and Related Health Problems, 10th Revision, German Modification*
IHE: Integrating the Healthcare Enterprise
ISO: International Organization for Standardization
ISO/IEC: International Organization for Standardization/International Electrotechnical Commission
LIS: laboratory information system
LOINC: Logical Observation Identifiers Names and Codes
MPI: Master Patient Index
MTB: Molecular Tumor Board
ORU: observation result unsolicited
PACS: picture archiving and communication system
PCM: Patient Consent Manager
PEHR: patient cross-enterprise health record
PGHD: patient-generated health data
RE: requirement engineering
RIS: radiology information system
UKSH: University Hospital Schleswig-Holstein
XDS: Cross-Enterprise Document Sharing
