# Supplementary material for: A Data-Centric Approach for Health Care and Research in a Health Knowledge Management Platform: Implementation and Requirement-Based Evaluation Study
Source: JMIR Med Inform. 2026 Apr 30;14:e83608. doi: 10.2196/83608 (PMC13131826; doi:10.2196/83608)
Supplement: Multimedia Appendix 1 [file medinform-v14-e83608-s001.pdf]

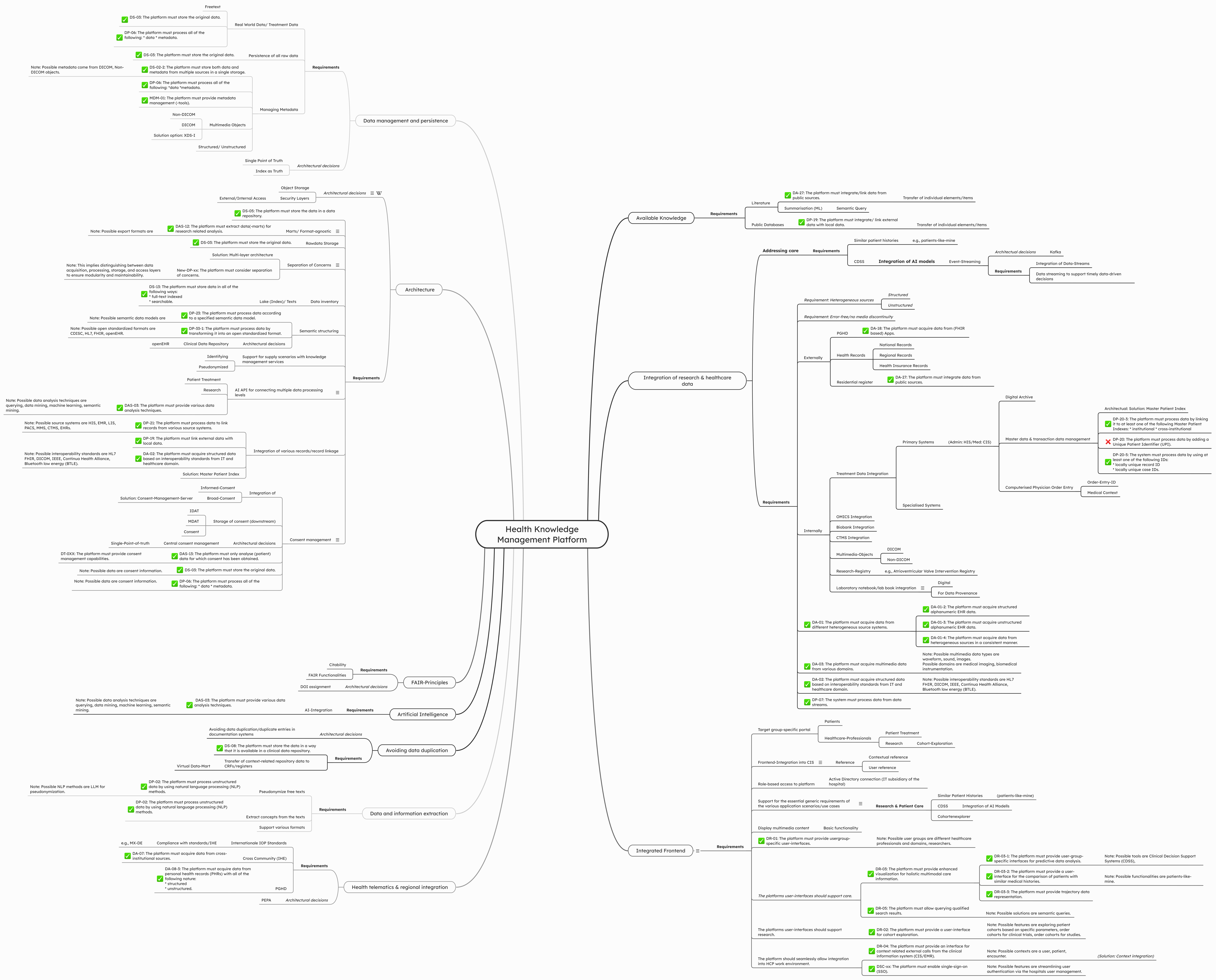
Health Knowledge Management Platform
Data management and persistence
Requirements
Real World Data/ Treatment Data
Freetext
DS-03: The platform must store the original data.
DP-06: The platform must process all of the following: * data * metadata.
Persistence of all raw data
DS-03: The platform must store the original data.
Managing Metadata
Note: Possible metadata come from DICOM, Non-DICOM objects.
DS-02-2: The platform must store both data and metadata from multiple sources in a single storage.
DP-06: The platform must process all of the following: *data *metadata.
MDM-01: The platform must provide metadata management (-tools).
Multimedia Objects
Non-DICOM
DICOM
Solution option: XDS-I
Structured/ Unstructured
Architectural decisions
Single Point of Truth
Index as Truth
Architecture
Architectural decisions
Object Storage
Security Layers
External/Internal Access
Requirements
DS-05: The platform must store the data in a data repository.
Marts/ Format-agnostic
DAS-12: The platform must extract data(-marts) for research related analysis.
Note: Possible export formats are
Rawdata Storage
DS-03: The platform must store the original data.
Separation of Concerns
Solution: Multi-layer architecture
New-DP-xx: The platform must consider separation of concerns.
Note: This implies distinguishing between data acquisition, processing, storage, and access layers to ensure modularity and maintainability.
Data inventory
Lake (Index)/ Texts
DS-13: The platform must store data in all of the following ways:
* full-text indexed
* searchable.
Semantic structuring
DP-23: The platform must process data according to a specified semantic data model.
Note: Possible semantic data models are
DP-33-1: The platform must process data by transforming it into an open standardized format.
Note: Possible open standardized formats are CDISC, HL7, FHIR, openEHR.
Architectural decisions
Clinical Data Repository
openEHR
Support for supply scenarios with knowledge management services
Identifying
Pseudonymized
AI API for connecting multiple data processing levels
Patient Treatment
Research
DAS-03: The platform must provide various data analysis techniques.
Note: Possible data analysis techniques are querying, data mining, machine learning, semantic mining.
Integration of various records/record linkage
DP-21: The platform must process data to link records from various source systems.
Note: Possible source systems are HIS, EMR, LIS, PACS, MMS, CTMS, EHRs.
DP-19: The platform must link external data with local data.
DA-02: The platform must acquire structured data based on interoperability standards from IT and healthcare domain.
Note: Possible interoperability standards are HL7 FHIR, DICOM, IEEE, Continua Health Alliance, Bluetooth low energy (BTLE).
Solution: Master Patient Index
Consent management
Integration of
Informed-Consent
Broad-Consent
Solution: Consent-Management-Server
Storage of consent (downstream)
IDAT
MDAT
Consent
Architectural decisions
Central consent management
Single-Point-of-truth
DAS-13: The platform must only analyse (patient) data for which consent has been obtained.
DT-0XX: The platform must provide consent management capabilities.
DS-03: The platform must store the original data.
Note: Possible data are consent information.
DP-06: The platform must process all of the following: * data * metadata.
Note: Possible data are consent information.
FAIR-Principles
Requirements
Citability
FAIR Functionalities
Architectural decisions
DOI assignment
Artificial Intelligence
Requirements
AI-Integration
DAS-03: The platform must provide various data analysis techniques.
Note: Possible data analysis techniques are querying, data mining, machine learning, semantic mining.
Avoiding data duplication
Architectural decisions
Avoiding data duplication/duplicate entries in documentation systems
Requirements
DS-08: The platform must store the data in a way that it is available in a clinical data repository.
Transfer of context-related repository data to CRFs/registers
Virtual Data-Mart
Data and information extraction
Requirements
Pseudonymize free texts
DP-02: The platform must process unstructured data by using natural language processing (NLP) methods.
Note: Possible NLP methods are LLM for pseudonymization.
Extract concepts from the texts
DP-02: The platform must process unstructured data by using natural language processing (NLP) methods.
Support various formats
Health telematics & regional integration
Requirements
Compliance with standards/IHE
e.g., MX-DE
Internationale IOP Standards
Cross Community (IHE)
DA-07: The platform must acquire data from cross-institutional sources.
PGHD
DA-08-3: The platform must acquire data from personal health records (PHRs) with all of the following nature:
* structured
* unstructured.
Architectural decisions
PEPA
Available Knowledge
Requirements
Literature
DA-27: The platform must integrate/link data from public sources.
Transfer of individual elements/items
Summarisation (ML)
Semantic Query
Public Databases
DP-19: The platform must integrate/ link external data with local data.
Transfer of individual elements/items
Integration of research & healthcare data
Addressing care
Requirements
Similar patient histories
e.g., patients-like-mine
CDSS
Integration of AI models
Event-Streaming
Architectual decisions
Kafka
Requirements
Integration of Data-Streams
Data streaming to support timely data-driven decisions
Requirements
Requirement: Heterogeneous sources
Structured
Unstructured
Requirement: Error-free/no media discontinuity
Externally
PGHD
DA-18: The platform must acquire data from (FHIR based) Apps.
Health Records
National Records
Regional Records
Health Insurance Records
Residential register
DA-27: The platform must integrate data from public sources.
Internally
Treatment Data Integration
Primary Systems
(Admin: HIS/Med: CIS)
Digital Archive
Master data & transaction data management
Architectual: Solution: Master Patient Index
DP-20-3: The platform must process data by linking it to at least one of the following Master Patient Indexes: * institutional * cross-institutional
DP-20: The platform must process data by adding a Unique Patient Identifier (UPI).
DP-20-5: The system must process data by using at least one of the following IDs:
* locally unique record ID
* locally unique case IDs.
Computerised Physician Order Entry
Order-Entry-ID
Medical Context
Specialised Systems
OMICS Integration
Biobank Integration
CTMS Integration
Multimedia-Objects
DICOM
Non-DICOM
Research-Registry
e.g., Atrioventricular Valve Intervention Registry
Laboratory notebook/lab book integration
Digital
For Data Provenance
DA-01: The platform must acquire data from different heterogeneous source systems.
DA-01-2: The platform must acquire structured alphanumeric EHR data.
DA-01-3: The platform must acquire unstructured alphanumeric EHR data.
DA-01-4: The platform must acquire data from heterogeneous sources in a consistent manner.
DA-03: The platform must acquire multimedia data from various domains.
Note: Possible multimedia data types are waveform, sound, images.
Possible domains are medical imaging, biomedical instrumentation.
DA-02: The platform must acquire structured data based on interoperability standards from IT and healthcare domain.
Note: Possible interoperability standards are HL7 FHIR, DICOM, IEEE, Continua Health Alliance, Bluetooth low energy (BTLE).
DP-07: The system must process data from data streams.
Integrated Frontend
Requirements
Target group-specific portal
Patients
Healthcare-Professionals
Patient Treatment
Research
Cohort-Exploration
Frontend-Integration into CIS
Reference
Contextual reference
User reference
Role-based access to platform
Active Directory connection (IT subsidiary of the hospital)
Support for the essential generic requirements of the various application scenarios/use cases
Research & Patient Care
Similar Patient Histories
(patients-like-mine)
CDSS
Integration of AI Modells
Cohortenexplorer
Display multimedia content
Basic functionality
DR-01: The platform must provide usergroup-specific user-interfaces.
Note: Possible user groups are different healthcare professionals and domains, researchers.
The platforms user-interfaces should support care.
DR-03: The platform must provide enhanced visualization for holistic multimodal care information.
DR-03-1: The platform must provide user-group-specific interfaces for predictive data analysis.
Note: Possible tools are Clinical Decision Support Systems (CDSS),
DR-03-2: The platform must provide a user-interface for the comparison of patients with similar medical histories.
Note: Possible functionalities are patients-like-mine.
DR-03-3: The platform must provide trajectory data representation.
DR-05: The platform must allow querying qualified search results.
Note: Possible solutions are semantic queries.
The platforms user-interfaces should support research.
DR-02: The platform must provide a user-interface for cohort exploration.
Note: Possible features are exploring patient cohorts based on specific parameters, order cohorts for clinical trials, order cohorts for studies.
The platform should seamlessly allow integration into HCP work environment.
DR-04: The platform must provide an interface for context related external calls from the clinical information system (CIS/EMR).
Note: Possible contexts are a user, patient, encounter.
(Solution: Context integration)
DSC-xx: The platform must enable single-sign-on (SSO).
Note: Possible features are streamlining user authentication via the hospitals user management.

Laboratory notebook/lab book integration

MII Sharepoint: AG-Metadaten 5 Sterne System für Trustability von Daten?! (HU)

Für Argumentation für Reihenfolge der Integration von (klinischen) Quellsystemen.

Integrated Frontend

Bisher wird nur Kohorten-Bezogen gearbeitet.

Live-Sicht auf die Daten aktuell nicht gewährleistet - aber gefragt.

Technisch möglich; rechtliches Problem das nur jeder das sieht was er darf - Forschung/ Versorgung.

Neue Anforderungen erstellen; bisher nicht im Katalog weil wir innovativ unterwegs sind.

Frontend-Integration into CIS

In Orbis: FRAU = Fremdaufruf

Support for the essential generic requirements of the various application scenarios/use cases

Zu unkonkret.

Architectural decisions

https://en.wikipedia.org/wiki/Separation_of_concerns

- Metadaten
- Terminologien
- Storage
-

Marts/ Format-agnostic

Projektbezogen zur Verfügung gestellter Export

Separation of Concerns

BS: Trennung der Zuständigkeiten - bspw. Freitexte in sicherer Umgebung/ Zugriff Kohortenexplorer nur auf strukturierte Daten.

Führt zur Architekturentscheidung: Mehrschichtige Architektur.

AI API for connecting multiple data processing levels

KI-API ist Architekturentscheidung

Consent management

Rechtsgrundlage für Bereitstellung notwendig
