# Supplementary material for: A Data-Centric Approach for Health Care and Research in a Health Knowledge Management Platform: Implementation and Requirement-Based Evaluation Study
Source: JMIR Med Inform. 2026 Apr 30;14:e83608. doi: 10.2196/83608 (PMC13131826; doi:10.2196/83608)
Supplement: Multimedia Appendix 2 [file medinform-v14-e83608-s002.docx]

#### Requirements Mapping Table for Health Knowledge Management Platform

**Table S1**. Alignment of platform requirements with architecture and scenarios. SrcL = Source Layer, AppL = Application Layer, SL, Staging Layer, NL = Normalization Layer, TBD = To be done, (✓) = partially fulfilled, ✓ = fulfilled

| *Req. ID* | *Layer* | *Description* | *Architecture* | *Cardio* | *Radio* | *Neuro* | *Source* |
| --- | --- | --- | --- | --- | --- | --- | --- |
| DA-01 | SrcL | The platform must acquire data from different heterogeneous source systems. | ✓ | ✓ | ✓ | ✓ | [2–6] |
| DA-01-2 | SrcL | The platform must acquire structured alphanumeric EHR data. | ✓ | ✓ | ✓ | n/a | [7] |
| DA-01-3 | SrcL | The platform must acquire unstructured alphanumeric EHR data. | ✓ | ✓ | n/a | n/a | [7] |
| DA-01-4 | SrcL | The platform must acquire data from heterogeneous sources in a consistent manner. | ✓ | ✓ | ✓ | ✓ | [5] |
| DA-02 | SrcL | The platform must acquire structured data based on interoperability standards from IT and healthcare domain. | ✓ | ✓ | ✓ | ✓ | [8–11] |
| DA-03 | SrcL | The platform must acquire multimedia data from various domains. | ✓ | n/a | ✓ | ✓ | [6,7,12] |
| DA-07 | SrcL | The platform must acquire data from cross-institutional sources. | ✓ | ✓ | n/a | n/a | [5] |
| DA-08-3 | SrcL | The platform must acquire data from personal health records (PHRs) with all of the following nature: * structured * unstructured. | ✓ | ✓ | n/a | n/a | [13,14] |
| DA-18 | AppL | The platform must acquire data from (FHIR based) Apps. | ✓ | ✓ | n/a | n/a | [15] |
| DA-27 | SrcL | The platform must integrate data from public sources. | TBD | n/a | n/a | n/a | [5,16] |
| DP-07 | SL | The platform must process data from data streams. | ✓ | ✓ | ✓ | n/a | [17] |
| DS-02-2 | SL | The platform must store both data and metadata from multiple sources in a single storage. | ✓ | ✓ | ✓ | ✓ | [18] |
| DS-03 | SL | The platform must store the original data. | ✓ | ✓ | ✓ | ✓ | [19,20] |
| DS-05 | NL | The platform must store data in a data repository. | ✓ | ✓ | ✓ | ✓ | [19] |
| DS-08 | NL | The platform must store the data in a way that it is available in a clinical data repository. | ✓ | ✓ | ✓ | n/a | [19] |
| DS-13 | SL | The platform must store data in all of the following ways:  * full-text indexed * searchable. | ✓ | ✓ | ✓ | ✓ | [21] |
| DP-01 | NL | The platform must process data by mapping it to multiple terminology standards. | ✓ | ✓ | ✓ | n/a | [4,7,7,10,14,17,21–32] |
| DP-02 | NL | The platform must process unstructured data by using natural language processing (NLP) methods. | TBD | n/a | n/a | n/a | [13,19,30–33] |
| DP-06 | NL | The platform must process all of the following: * data * metadata. | ✓ | ✓ | ✓ | ✓ | [10,34] |
| MDM-01 | NL | The platform must provide metadata management(-tools). | ✓ | n/a | ✓ | n/a | [33,35] |
| DP-19 | NL | The platform must link external data with local data. | (✓) | ✓ | n/a | n/a | [36] |
| DP-20-3 | NL | The platform must process data by linking it to at least one of the following Master Patient Indexes: * institutional * cross-institutional. | ✓ | ✓ | ✓ | n/a | [21,37] |
| DP-20-5 | NL | The platform must process data by using at least one of the following IDs:  * locally unique record ID * locally unique case IDs. | ✓ | ✓ | ✓ | n/a | [37] |
| DP-21 | NL | The platform must process data to link records from various source systems. | ✓ | ✓ | ✓ | n/a | [11] |
| DP-23 | NL | The platform must process data according to a specified semantic data model. | ✓ | ✓ | ✓ | ✓ | [7,14,31] |
| DP-33-1 | NL | The platform must process data by transforming it into an open standardized format. | ✓ | ✓ | ✓ | ✓ | [26,30,38] |
| DSC-04 | NL | The platform must provide a pseudonymization ID (PID) to disconnect medical data from identifying data. | ✓ | ✓ | ✓ | ✓ | [30] |
| DAS-03 | AL | The platform must provide various data analysis techniques. | TBD | n/a | n/a | n/a | [35] |
| DAS-12 | AL | The platform must extract data(-marts) for research related analysis. | ✓ | ✓ | ✓ | ✓ | [39] |
| DAS-13 | NL | The platform must only analyse (patient-) data for which consent has been obtained. | ✓ | n/a | ✓ | n/a | [39] |
| DR-01 | AppL | The platform must provide user-group-specific user-interfaces. | ✓ | ✓ | n/a | n/a | additional RE |
| DR-02 | AppL | The platform must provide a user-interface for cohort exploration. | ✓ | n/a | n/a | n/a | additional RE |
| DR-03 | AppL | The platform must provide enhanced visualization for holistic multimodal care information. | TBD | n/a | n/a | n/a | additional RE |
| DR-03-1 | AppL | The platform must provide user-group-specific interfaces for predictive data analysis. | TBD | n/a | n/a | n/a | additional RE |
| DR-03-2 | AppL | The platform must provide a user-interface for the comparison of patients with similar medical histories. | TBD | n/a | n/a | n/a | additional RE |
| DR-03-3 | AppL | The platform must provide trajectory data representation. | ✓ | n/a | n/a | n/a | additional RE |
| DR-04 | AppL | The platform must provide an interface for context related external calls from the clinical information system (CIS/EMR). | TBD | n/a | n/a | n/a | additional RE |
| DR-05 | AppL | The platform must allow querying qualified search results. | ✓ | ✓ | ✓ | n/a | additional RE |
| DSC-12 | AppL | The platform must enable single-sign-on (SSO). | (✓) | n/a | n/a | n/a | additional RE |

#### Bio signals in a neurology scenario

The neurology scenario focused on EEG data integration for open science research, adopting a one-time legacy data integration approach for the purpose of research data management. The EEG data set utilized in this study is provided by the Center for Integrative Psychiatry's Sleep Laboratory and the Department of Neurology, both at USKH. The data were recorded using two different mobile EEG device "Dreem 2" and a “V-Amp” with goldcup scalp electrodes. Each device exports data in a different proprietary data format.

The platform's task was to acquire and store EEG data from both devices (DS-02-2, DS-03), harmonize them, and provide them to a third party for training a de-identification algorithm. Initially, a legacy import of 60 data sets was done, which required transforming the raw data into the Brain Imaging Data Structure (BIDS) format (DA-01, DA-02, DA-03, DP-23) [1]. By adopting the BIDS format, we enhance the quality assurance by promoting standardized data organization and metadata specifications (DP-06, DA-01-4), thus ensuring consistency and reproducibility in neuroimaging research. The transformation process involved organizing, annotating and describing the data in accordance with the BIDS standard (DP-33-1). Adhering to this structure across non-DICOM multimedia data sets ensure standardized and interoperable data, facilitating seamless integration and analysis within the platform (DA-02, DA-03). Afterwards, EEG data were harmonized and made available for further processing. The platform ensures consistent naming conventions, units of measurement, and data organization principles, enabling interpretation and analysis. Furthermore, the EEG data set in the BIDS format, is securely stored in S3 and indexed in Elasticsearch, ensuring findability, accessibility, interoperability and re-use (FAIR data) (DS-05, DS-13). In addition, the standardized metadata using BIDS in combination with MPI and consent integration allowed a reliable removal of identifying information such as the patients’ names and identifier (DSC-04) from the harmonized EEG data before transferring to the third party. The final data set was exported for sharing with the project partners (DAS-12).

#### Requirements References

1. Gorgolewski KJ, Auer T, Calhoun VD, Craddock RC, Das S, Duff EP, Flandin G, Ghosh SS, Glatard T, Halchenko YO, Handwerker DA, Hanke M, Keator D, Li X, Michael Z, Maumet C, Nichols BN, Nichols TE, Pellman J, Poline J-B, Rokem A, Schaefer G, Sochat V, Triplett W, Turner JA, Varoquaux G, Poldrack RA. The brain imaging data structure, a format for organizing and describing outputs of neuroimaging experiments. Sci Data 2016 June 21;3(1):160044. doi: 10.1038/sdata.2016.44

2. Ethier J-F, McGilchrist M, Barton A, Cloutier A-M, Curcin V, Delaney BC, Burgun A. The TRANSFoRm project. Learn Health Syst 2018;2(2):e10037. doi: 10.1002/lrh2.10037

3. Lelong R, Soualmia LF, Grosjean J, Taalba M, Darmoni SJ. Building a Semantic Health Data Warehouse in the Context of Clinical Trials. JMIR Med Inform 2019;7(4):153–169. doi: 10.2196/13917

4. Tahar K, Müller C, Dürschmid A, Haferkamp S, Saleh K, Jürs P, Stäubert S, Gewehr JE, Zenker S, Ammon D, Wendt T. Integrating Heterogeneous Data Sources for Cross- Institutional Data Sharing. Stud Health Technol Inform IOS Press; 2019;264:1785–1786. doi: 10.3233/SHTI190647

5. Tsiknakis M, Rueping S, Martin L, Sfakianakis S, Bucur A, Sengstag T, Brochhausen M, Pucaski J, Graf N. Developing a European grid infrastructure for cancer research. Ecancermedicalscience 2007;1:56. doi: 10.3332/ecms.2007.56

6. Gupta A, Ludascher B, Martone ME. Knowledge-based integration of neuroscience data sources. Berlin, Germany: IEEE Computer Society Press; 2000. p. 39–52. doi: 10.1109/SSDM.2000.869777

7. Katehakis DG, Sfakianakis SG, Kavlentakis G, Anthoulakis DN, Tsiknakis M. Delivering a Lifelong Integrated Electronic Health Record Based on a Service Oriented Architecture. IEEE Trans Inform Technol Biomed 2007;11(6):639–650. doi: 10.1109/TITB.2006.889711

8. Urbauer P, Kmenta M, Frohner M, Mense A, Sauermann S. Propose of Standards Based IT Architecture to Enrich the Value of Allergy Data by Telemonitoring Data..."Health Informatics meets eHealth conference," Vienna, Austria, 2017. Stud Health Technol Inform IOS Press; 2017;236:136–143. doi: 10.3233/978-1-61499-759-7-136

9. Katehakis DG, Tsiknakis M, Orphanoudakis SC. A healthcare information infrastructure to support integrated services over regional health telematics networks. Heahtl IT Advis Rep Boston, Massachusetts: Medical Records Institute; 2002;3(1):15–18. doi: 10.1109/IEMBS.2001.1019624

10. Firnkorn D, Ganzinger M, Muley T, Thomas M, Knaup P. A Generic Data Harmonization Process for Cross-linked Research and Network Interaction. Construction and Application for the Lung Cancer Phenotype Database of the German Center for Lung Research. Methods Inf Med Schattauer GmbH; 2015;54(5):455–460. doi: 10.3414/ME14-02-0030

11. Haak D, Page C-E, Reinartz S, Krüger T, Deserno TM. DICOM for Clinical Research. J Digit Imaging 2015;28(5):558–566. doi: 10.1007/s10278-015-9802-8

12. Rajasekaran H, Lo Iacono L, Hasselmeyer P, Fingberg J, Summers P, Benkner S, Engelbrecht G, Arbona A, Chiarini A, Friedrich CM, Hofmann-Apitius M, Kumpf K, Moore B, Bijlenga P, Iavindrasana J, Mueller H, Hose RD, Dunlop R, Frangi AF, editors. @neurIST - Towards a system architecture for advanced disease management through integration of heterogeneous data, computing, and complex processing services. 2008. doi: 10.1109/CBMS.2008.42

13. Botsis T, Hartvigsen G, Chen F, Weng C. Secondary Use of EHR. Summit on translational bioinformatics 2010;2010:1–5.

14. Marschollek M, Wolf K-H, Bott O-J, Geisler M, Plischke M, Ludwig W, Hornberger A, Haux R. Sustainable ubiquitous home health care--architectural considerations and first practical experiences. Stud Health Technol Inform 2007;129(Pt 1):8–12.

15. Warner JL, Jain SK, Levy MA. Integrating cancer genomic data into electronic health records. Genome medicine 2016;8(1):113. doi: 10.1186/s13073-016-0371-3

16. Teodoro D, Pasche E, Gobeill J, Emonet S, Ruch P, Lovis C. Building a transnational biosurveillance network using semantic web technologies: requirements, design, and preliminary evaluation. Journal of Medical Internet Research Toronto, Ontario: JMIR Publications Inc.; 2012 Aug 7;14(4):e73–e73.

17. Denecke K. Integrating Social Media and Mobile Sensor Data for Clinical Decision Support: Concept and Requirements. Studies in health technology and informatics 2016;225:562–566.

18. Ozaydin B, Zengul F, Oner N, Feldman SS. Healthcare Research and Analytics Data Infrastructure Solution: A Data Warehouse for Health Services Research. J Med Internet Res Toronto: Jmir Publications, Inc; 2020 June 4;22(6):e18579. doi: 10.2196/18579

19. Bouzillé G, Westerlynck R, Defossez G, Bouslimi D, Bayat S, Riou C, Busnel Y, Le Guillou C, Cauvin J-M, Jacquelinet C, Pladys P, Oger E, Stindel E, Ingrand P, Coatrieux G, Cuggia M. Sharing Health Big Data for Research - A Design by Use Cases. Stud Health Technol Inform IOS Press; 2017;245:303–307. doi: 10.3233/978-1-61499-830-3-303

20. Ohmann C, Kuchinke W. Future Developments of Medical Informatics from the Viewpoint of Networked Clinical Research Interoperability and Integration. Methods Inf Med 2009;48(1):45–54. doi: du

21. Lowe HJ, Ferris TA, Hernandez PM, Weber SC. STRIDE--An integrated standards-based translational research informatics platform. AMIA . Annual Symposium proceedings AMIA Symposium 2009;2009:391–395.

22. Sujansky W. Heterogeneous Database Integration in Biomedicine. Journal of Biomedical Informatics 2001;34(4):285–298. doi: 10.1006/jbin.2001.1024

23. Bestek M, Stanimirovic D. Special Topic Interoperability and EHR: Combining openEHR, SNOMED, IHE, and Continua as approaches to interoperability on national eHealth. Applied Clinical Informatics 2017;8(3):810–825. doi: 10.4338/ACI-2017-01-RA-0011

24. Ayatollahi H, Hosseini SF, Hemmat M. Integrating Genetic Data into Electronic Health Records: Medical Geneticists’ Perspectives. Healthcare Informatics Research 2019 Oct;25(4):289–296. doi: 10.4258/hir.2019.25.4.289

25. Daniel C, Ouagne D, Sadou E, Paris N, Hussain S, Jaulent M-C, Kalra D. Cross border semantic interoperability for learning health systems: The EHR4CR semantic resources and services. Learning Health Systems 2017 Jan;1(1):e10014. doi: 10.1002/lrh2.10014

26. Deserno T, Haak D, Brandenburg V, Deserno V, Classen C, Specht P. Integrated Image Data and Medical Record Management for Rare Disease Registries. A General Framework and its Instantiation to the German Calciphylaxis Registry. Journal of Digital Imaging ,: Springer Nature; 2014 Dec;27(6):702–713. doi: 10.1007/s10278-014-9698-8

27. Ethier J-F, Curcin V, McGilchrist MM, Choi Keung SNL, Zhao L, Andreasson A, Bródka P, Michalski R, Arvanitis TN, Mastellos N, Burgun A, Delaney BC. eSource for clinical trials. Int J Med Inform New York, New York: Elsevier B.V; 2017;106:17–24. doi: 10.1016/j.ijmedinf.2017.06.006

28. Ethier J-F, Dameron O, Curcin V, McGilchrist MM, Verheij RA, Arvanitis TN, Taweel A, Delaney BC, Burgun A. A unified structural/terminological interoperability framework based on LexEVS. J Am Med Inform Assoc Oxford University Press / USA; 2013;20(5):986–994. doi: 10.1136/amiajnl-2012-001312

29. Oliveira IC, Oliveira JL, Sanchez JP, López-Alonso V, Martin-Sanchez F, Maojo V, Sousa Pereira A. Grid requirements for the integration of biomedical information resources for health applications. Methods Inf Med 2005;44(2):161–167.

30. Rance B, Canuel V, Countouris H, Laurent-Puig P, Burgun A. Integrating Heterogeneous Bio-medical Data for Cancer Research: the CARPEM infrastructure. Applied Clinical Informatics 2016;7(2):260–274. doi: 10.4338/ACI-2015-09-RA-0125

31. Chute CG, Beck SA, Fisk TB, Mohr DN. The Enterprise Data Trust at Mayo Clinic. Journal of the American Medical Informatics Association 2010;17(2):131–135. doi: 10.1136/jamia.2009.002691

32. Hall JL, Ryan JJ, Bray BE, Brown C, Lanfear D, Newby LK, Relling MV, Risch NJ, Roden DM, Shaw SY, Tcheng JE, Tenenbaum J, Wang TN, Weintraub WS. Merging Electronic Health Record Data and Genomics for Cardiovascular Research. Circulation Cardiovascular genetics 2016;9(2):193–202. doi: 10.1161/HCG.0000000000000029

33. Mo H, Thompson WK, Rasmussen LV, Pacheco JA, Jiang G, Kiefer R, Zhu Q, Xu J, Montague E, Carrell DS, Lingren T, Mentch FD, Ni Y, Wehbe FH, Peissig PL, Tromp G, Larson EB, Chute CG, Pathak J, Denny JC, Speltz P, Kho AN, Jarvik GP, Bejan CA, Williams MS, Borthwick K, Kitchner TE, Roden DM, Harris PA. Desiderata for computable representations of electronic health records-driven phenotype algorithms. Journal of the American Medical Informatics Association 2015;22(6):1220–1230. doi: 10.1093/jamia/ocv112

34. Karasavvas KA, Baldock R, Burger A. Bioinformatics integration and agent technology. Journal of Biomedical Informatics 2004;37(3):205–219. doi: 10.1016/j.jbi.2004.04.003

35. Hackl WO, Ammenwerth E. SPIRIT: Systematic Planning of Intelligent Reuse of Integrated Clinical Routine Data. A Conceptual Best-practice Framework and Procedure Model. Methods of Information in Medicine Schattauer GmbH; 2016 Mar;55(2):114–124. doi: 10.3414/ME15-01-0045

36. Fillinger S, de la Garza L, Peltzer A, Kohlbacher O, Nahnsen S. Challenges of big data integration in the life sciences. Anal Bioanal Chem 2019 Oct 1;411(26):6791–6800. doi: 10.1007/s00216-019-02074-9

37. Al Jarullah A, El-Masri S. Proposal of an architecture for the national integration of Electronic Health Records: A semi-centralized approach. Studies in health technology and informatics 2012;180:917–921.

38. Hanss S, Wetzel T, Schaaf T, Hahn C, Schrader T, Tolxdorff T. Integration of decentralized clinical data in a data warehouse. Methods Inf Med Schattauer GmbH; 2009;48(5):414–418. doi: 10.3414/ME9240

39. Bouzillé Guillaume, Sylvestre Emmanuelle, Campillo-Gimenez Boris, Renault Eric, Ledieu Thibault, Delamarre Denis, Cuggia Marc. An Integrated Workflow For Secondary Use of Patient Data for Clinical Research. Studies in Health Technology and Informatics IOS Press; 2015;216:913. doi: 10.3233/978-1-61499-564-7-913
